# eNOS controls angiogenic sprouting and retinal neovascularization through the regulation of endothelial cell polarity

**DOI:** 10.1007/s00018-021-04042-y

**Published:** 2021-12-31

**Authors:** Tracy L. Smith, Malika Oubaha, Gael Cagnone, Cécile Boscher, Jin Sung Kim, Yassine El Bakkouri, Ying Zhang, Rony Chidiac, Jeanne Corriveau, Chantal Delisle, Gregor U. Andelfinger, Przemyslaw Sapieha, Jean-Sébastien Joyal, Jean-Philippe Gratton

**Affiliations:** 1 Department of Pharmacology and Physiology, Faculty of Medicine, Université de Montréal, Montreal, QC Canada; 2 Département des Sciences Biologiques, Université du Québec à Montréal (UQAM), Montreal, QC Canada; 3 Department of Pediatrics and Centre Hospitalier Universitaire Ste-Justine, Université de Montréal, Montreal, QC Canada; 4 School of Pharmaceutical Sciences, Tsinghua University, Beijing, China; 5 Donnelly Centre, University of Toronto, Toronto, Canada; 6 Department of Ophthalmology, Maisonneuve-Rosemont Hospital Research Centre, Université de Montréal, Montreal, QC Canada

**Keywords:** Nitric oxide, VEGF, Angiogenesis, Polarity, Retina, Retinopathies, Tip-cells

## Abstract

The roles of nitric oxide (NO) and endothelial NO synthase (eNOS) in the regulation of angiogenesis are well documented. However, the involvement of eNOS in the sprouting of endothelial tip-cells at the vascular front during sprouting angiogenesis remains poorly defined. In this study, we show that downregulation of eNOS markedly inhibits VEGF-stimulated migration of endothelial cells but increases their polarization, as evidenced by the reorientation of the Golgi in migrating monolayers and by the fewer filopodia on tip cells at ends of sprouts in endothelial cell spheroids. The effect of eNOS inhibition on EC polarization was prevented in Par3-depleted cells. Importantly, downregulation of eNOS increased the expression of polarity genes, such as PARD3B, PARD6A, PARD6B, PKCΖ, TJP3, and CRB1 in endothelial cells. In retinas of eNOS knockout mice, vascular development is retarded with decreased vessel density and vascular branching. Furthermore, tip cells at the extremities of the vascular front have a marked reduction in the number of filopodia per cell and are more oriented. In a model of oxygen-induced retinopathy (OIR), eNOS deficient mice are protected during the initial vaso-obliterative phase, have reduced pathological neovascularization, and retinal endothelial tip cells have fewer filopodia. Single-cell RNA sequencing of endothelial cells from OIR retinas revealed enrichment of genes related to cell polarity in the endothelial tip-cell subtype of eNOS deficient mice. These results indicate that inhibition of eNOS alters the polarity program of endothelial cells, which increases cell polarization, regulates sprouting angiogenesis and normalizes pathological neovascularization during retinopathy.

**Supplementary Information:**

The online version contains supplementary material available at 10.1007/s00018-021-04042-y.

## Introduction

Angiogenesis, the process by which new blood vessels arise from pre-existing vessels, is a multistep process that occurs in response to external cues such as Vascular Endothelial Growth Factor (VEGF) and physical factors such as cell–cell and cell–matrix interactions. During sprouting angiogenesis, ECs differentiate into highly motile tip-cells and proliferative stalk cells, a process that is primarily regulated by Notch-Dll4-VEGF pathways [1, 2]. Tip-cells extend filopodia and branch out directionally in response to guidance cues and adopt a polarized morphology [3], while stalk-cells maintain cell–cell contacts during sprouting to preserve vessel integrity and form lumens for blood perfusion [4]. During this collective cell migration process, it is necessary that tip-cells at the front adopt a polarized morphology and that cell–cell contacts at the back of endothelial sprouts are maintained [3]. The production of nitric oxide (NO) by endothelial NO synthase (eNOS) is essential for the induction of angiogenesis and vascular permeability induced by VEGF and eNOS-derived NO has been shown to promote survival and migration of endothelial cells (ECs) [5–9].

Angiogenesis is essential during embryonic development and other physiological events such as wound healing but can also be a driving factor in several pathologies including ischemic retinopathies such as retinopathy of prematurity (ROP), a major complication of preterm birth that can lead to blindness. Several factors can contribute to ROP [10]. One of the strongest risk factors in the genesis of ROP is high postnatal oxygenation [11–13]. Premature infants are exposed to higher levels of oxygen tension after birth compared to what they would usually be exposed to at this stage of development in utero, especially if the infant requires oxygen supplementation; this leads to suppression of VEGF [14, 15]. In development, delicate growing retinal capillaries are overly sensitive to oxygen [16]. The relative hyperoxia leads to the initial vaso-obliteration phase of ROP, wherein normal retinal vascular development stops and the existing microvasculature of the retina regresses following EC death [15, 17]. As the retina develops its metabolic demand increases leading to a higher requirement of blood supply than the depleted microvascular can provide [18]. The retina becomes hypoxic, triggering the release of VEGF and initiating the second vaso-proliferative phase of ROP. The vaso-proliferative phase is characterized by hypoxia-induced compensatory pathological neovascularization with poorly patterned and abnormally leaky vessels, which can lead to the formation of fibrous scarring and in severe cases retinal detachment and blindness [17–19]. eNOS knockout mice appear to be protected from the initial oxygen-induced vaso-obliteration and display reduced pathological neovascularization after hyperoxia [20]. Conversely, transgenic overexpression of eNOS in mice increased vascular closure in the obliterative phase and exacerbated the formation of neovascular tufts [21]. The role of eNOS-derived NO in the augmentation of vascular permeability to blood macromolecules is well established [5] and vascular leakage is central to the abnormalities associated with pathological neovascularization in retinopathies [22]. In addition, NO can directly promote disassembly of adherens junction in ECs, which increases endothelial permeability. Indeed, eNOS-derived NO can S-nitrosylate the adherens junction protein β-catenin to induce dissociation from VE-cadherin and eNOS deficiency in mice reduced VEGF-induced VE-cadherin phosphorylation preventing disassembly of adherens junctions [23, 24]. A recent study revealed that NO contributes to vascular hyperpermeability during retinopathy in mice by promoting tyrosine phosphorylation of VE-cadherin and destabilization of adherens junctions [25].

While the molecular contribution of NO and eNOS to vascular permeability and angiogenesis is well investigated [26], their contribution to tip-cell polarization during EC sprouting remains unresolved. Considering the importance of tip-cells and the demonstrated importance of eNOS-derived NO for migration of EC, defining the effects of eNOS on the activity of endothelial tip-cells in the retina and their speed of migration at the vascular front is of paramount importance. This could reveal a role for eNOS-derived NO in sprouting angiogenesis in the retina. Herein, we demonstrate that eNOS participates in the polarization program in ECs which influences their migration and angiogenic sprouting. Furthermore, we demonstrate a role for eNOS in endothelial tip-cells during vascular development of the retina in mice and during both the initial vaso-obliterative phase and subsequent neovascularization stage of oxygen-induced retinopathy. Moreover, we provide insights into the effects of eNOS on the transcriptional program of ECs affecting cell polarization during the neovascularization stage of OIR using a single cell RNA-sequencing approach. Thus, this study highlights the importance of eNOS on EC polarization during cell migration, which has consequences during sprouting angiogenesis in normal and pathological settings.

## Materials and methods

### Cell culture

Bovine Aortic Endothelial Cells (BAECs) (VEC Technologies, Rensselaer, NY, USA), selected for their ease of transfections and strong migratory responses to VEGF stimulation, were cultured in Dulbecco modified Eagle medium (DMEM) supplemented with 10% fetal bovine serum (HyClone Laboratories), 2.0 mM l-glutamine, 100 U/mL penicillin, and 100 μg/mL streptomycin.

Microvascular mouse lung endothelial cells (MLECs) were isolated from the lungs of eNOS^+/+^ and eNOS^−/−^ mice as previously described [27]. MLEC were grown in DMEM:F12 (1:1) medium (Gibco, Thermo Fisher Scientific, Waltham, MA, USA), containing 20% FBS (HyClone, GE Healthcare Life Sciences, Piscataway, NJ, USA), 0.05 mg/mL Endothelial cell growth supplement (Corning Life Science, Tewksbury, MA, USA), 0.1 mg/mL heparin (Millipore-Sigma, St-Louis, MO, USA), 100 U/mL penicillin and 100 μg/mL streptomycin (Thermo Fisher Scientific) and plated in 0.1% gelatin-coated T75 flasks. Magnetic Dynabeads (Thermo Fisher Scientific) were conjugated with anti-mouse CD102 (clone 3C4; BD Biosciences, Franklin Lakes, NJ, USA) antibody. Beads were added to each flask and incubated for 1 h at 4 °C. Cells were subsequently selected in a magnetic field for 10 min following trypsinization.

### Transfections and cell treatments

Control small interfering RNA (siRNA) and siRNA against eNOS were generated by Horizon Discoveries (Chicago, IL, USA). Sequences were designed against bovine eNOS [*NOS3*]: 5′-CCAGGAAGAAGACCUUUAAUU-3′, 5′- CCAACAUGCUGCUGGAAAUUU-3′, and against bovine Par3 [*PARD3*]: 5′-GGAAAGGAUUCA AGCCAAA-3′ as well as a non-related scrambled siRNA used as a control: 5′- AUGAACGUGAAUUGCUCAAUU-3′. BAECs were transfected with duplexes of RNA using Lipofectamine 2000 in OptiMEM (Thermo Fisher Scientific). Cells at 80–90% confluency were transfected with 50 mM of control or eNOS and/or Par3 siRNA for 48 h. Cells were then serum-starved for 6 h before treatment with recombinant human VEGF-A (40 ng/mL) for an additional 6 h (R&D Systems, Minneapolis, MN, USA).

### 3-Dimensional sprouting assay

Spheroids were prepared as previously described [28]. Briefly, transfected BAECs were cultured in DMEM containing 0.3% methylcellulose (Sigma-Aldrich), 10% FBS, 1% l-glutamine, and 1% penicillin/streptomycin in U-shaped 96-well plates for 24 h to allow spheroid formation. Spheroids were transferred in complete medium containing 50% collagen (Corning, Corning, NY, USA) pH 7.4, and 0.6% methylcellulose and cultured for 24 h before fixation in 4% PFA. Image acquisitions were performed using an inverted Axio-observer Z1 epifluorescent microscope (Zeiss, Germany) with a 20 × objective. The numbers of sprouts and filopodia lengths were determined manually using ZEN Blue 2.3 (Zeiss). Filopodia were defined as actin-rich extensions from the tip-cells of the sprout with < 0.1 μm diameter. For length quantification, only filopodia visible in one focus plane were manually counted by drawing lines from the bases of the cells to the tips of the filopodia using the line tool of ZEN Blue.

### Wound healing migration assay and time-lapse video microscopy

BAECs transfected with siRNAs were plated in 24-well plates and allowed to reach 90% confluency over 48 h. Confluent cells were incubated with the fluorescent vital Hoechst dye (Thermo Fisher Scientific) for 10 min before scratches were performed with a 200 μL pipette tip on the monolayer. Cell movement in the presence or absence of 40 ng/mL VEGF was recorded using an Axio-Observer Z1 epifluorescence microscope (Zeiss) equipped with an AxioCam MRm camera (Zeiss) and programmed to capture a frame every 10 min of the migration period (6 h). The temperature was maintained at 37 °C and the atmosphere within the chamber (PECON, Germany) was kept at 5% CO_2_/95% air throughout the experiment. Nuclei of the leading-edge cells were tracked by time-lapse video microscopy using the Cell Tracker plug-in of ImageJ (NIH). Nucleus tracks were analyzed with the Track Manager plug-in of Icy—OpenSource Image Processing Software. The total displacement, net displacement, and persistence were obtained for each track, and statistical analysis was performed.

### Antibodies and immunoblotting

Cells were solubilized with a lysis buffer containing 1% Nonidet P-40, 0.1% sodium dodecyl sulfate (SDS), 0.1% deoxycholic acid, 50 mM Tris–HCl (pH 7.4), 150 mM NaCl, 0.1 mM ethylenediaminetetraacetic acid (EDTA), 0.1 mM ethylene glycol tetraacetic acid (EGTA), 20 mM sodium fluoride, 1 mM sodium pyrophosphate, and 1 mM sodium orthovanadate and Complete mini EDTA free protease inhibitor cocktail (Roche Diagnostics, Indianapolis, IN, USA). Soluble proteins were separated by SDS-PAGE, transferred onto a nitrocellulose membrane (Hybond-ECL, GE Healthcare Life Science), and western blotted. Detection and quantification were performed using HRP-coupled antibodies and an Image Quant LAS4000 chemiluminescence-based detection system (enhanced chemiluminescence) (GE Healthcare Life Science).

Primary antibodies used for immunoblotting were mouse anti-eNOS (BD Biosciences, San Jose, CA, USA), mouse anti-β-Actin (New England Laboratories, Danvers, MA) and horseradish peroxidase (HRP)-coupled secondary antibodies Jackson ImmunoResearch Laboratories (West Grove, PA, USA). For Golgi immunofluorescence staining, anti-GM130 (BD Biosciences) and Alexa-coupled secondary antibodies from Thermo Fisher Scientific were used.

### Immunofluorescence analyses

Cells were cultured on 0.1% gelatin-coated coverslips and transfected as previously described. Cells were serum-starved overnight (BAECs) or for 6 h (MLECs) and stimulated with VEGF (40 ng/mL). Cells were then fixed in 4% PFA before permeabilization in 0.1% Triton for 5 min and blocked in 1% bovine serum albumin (BSA). Primary and secondary antibodies were incubated on fixed cells in 1% BSA-PBS (phosphate-buffered saline) solution for 1 h before being mounted in Fluoromount (Sigma-Aldrich). Acquisitions were performed on an LSM 800 confocal laser-scanning microscope (Zeiss).

To quantify cell orientation, BAECs were transfected with siRNA and cultured for 48 h until confluence. Scratches on BAECs and MLECs monolayers were performed with 200 μL tips, rinsed, and left to equilibrate for 15 min in serum-free medium. Cells were then stimulated with VEGF (40 ng/mL) for 30 min. Cells were then fixed with 4% PFA and nuclei (4′,6-diamidino-2-phenylindole [DAPI]; Sigma-Aldrich), Golgi (GM130) and F-actin (phalloidin) were stained. A total of 8–10 fields were quantified for each condition, representing 150–200 cells, and each experiment was repeated three times.

### RNA extraction and quantitative RT-PCR (qRT-PCR)

Total RNA was extracted and suspended in commercial RNase-free water with a RNeasy Minikit (Qiagen, Germany). After DNase I treatment, cDNA was synthesized from 1 µg total RNA using the SuperScript II Reverse Transcriptase kit for RT-PCR (Thermo Fisher Scientific) according to the manufacturer’s instructions. cDNA (5 ng) was amplified in triplicate using SYBR Select Master Mix or Power Up SYBR Master Mix (Thermo Fisher Scientific). Quantitative real-time PCR was performed with ViiATM 7 Real-Time PCR System (Thermo Fisher Scientific) or a MIC qPCR cycler (Bio Molecular Systems). Gene expression analysis was performed using the comparative cycle threshold (ΔCT) method, normalized with the expression of reference genes β-actin and GAPDH and presented as the mean fold change (± SEM) compared with control samples.

### RNA seq analysis

The RNA samples were submitted to IRCM (Institut de recherches cliniques de Montréal) molecular biology core facility. The mRNA was captured using poly(T)-coated magnetic beads followed by reverse transcription to generate the cDNA library using the TruSeq Stranded mRNA (Illumina, CA, USA). The whole transcriptome sequencing was done at Genome Quebec using an Illumina sequencer Hiseq2000.

The reads from RNA-seq were aligned to the Bos Taurus reference genome UMD 3.1, with TopHat using the Ensembl annotation provided with the Illumina iGenomes. Htseq-count script (v0.5.3) was used to count the number of reads aligned to each gene. The gene expression levels were calculated with Cufflinks (v2.1.1), a software that estimates the abundance of transcripts and tests for differential expression in RNA-Seq samples [29]. The counts were normalized relative to the sequencing depth with DESeq (v1.12.0).

In each experimental condition, genes up-/down-regulated more than 2 folds (> 2 or < 0.5 fold; >1 or < − 1 log2 fold) in the treatment condition comparing to the control condition were defined as regulated genes. The lists of regulated genes were further submitted for bioinformatics analysis. Bioinformatics analysis was performed using DAVID v6.7 (the Database for Annotation, Visualization and Integrated Discovery), and the enrichment of gene ontology (GO) categories and Kyoto Encyclopedia of Genes and Genomes (KEGG) pathway was analyzed. The submitted gene IDs were converted from bovine Ensemble ID to human Ensemble ID since the human database contains a more extensive list of characterized genes.

Each identifier was mapped to its corresponding objects provided by DAVID database. In the biological process analyses, cellular components and molecular function, the GO terms ‘GOTERM_BP_FAT’, ‘GOTERM_CC_FAT’ and ‘GOTERM_MF_FAT’ were applied. EASE score, a conservative adjustment to the Fisher exact probability, was used to calculate the Modified Fisher Exact *p*-value. The enrichment of GO terms was considered significant when the *p*-value is < 0.05.

### Animals

All studies were performed according to the Association for Research in Vision and Ophthalmology (ARVO) Statement for the Use of Animals in Ophthalmic and Vision Research and were approved by the Animal Care Committee of the University of Montreal in agreement with the guidelines established by the Canadian Council on Animal Care. C57BL/6J wildtype and mice homozygous for the nitric oxide synthase 3 (*Nos3*) knockout allele (eNOS^−/−^) (strain no. 002684; backcrossed to C57BL/6J for 15 generations) were purchased from The Jackson Laboratory (Bar Harbor, ME, USA).

### Model of oxygen-induced retinopathy (OIR)

Mouse pups (eNOS^+/+^ or eNOS^−/−^) and their fostering mothers (CD1, Charles River, Wilmington, MA, USA) were exposed to 75% O_2_ from P7 to P12 and returned to room air (normoxia) [19] using an oxygen chamber (BioSpherix, Parish, NY, USA). Eyes were enucleated at different time points and fixed in 4% paraformaldehyde (PFA). Retinas were then directly dissected for flat-mount.

### Immunohistochemistry of retinas

Dissection and whole-mount staining of postnatal retinas were performed as described previously [39]. Retinas were fixed for 2 h on ice in 4% paraformaldehyde and blocked overnight in 1% bovine serum albumin, 0.3% Triton X-100 in phosphate-buffered saline (PBS). Pan-retinal vasculature was visualized by isolectin B4 staining. Retinas were equilibrated in 1 mM CaCl_2_, 1 mM MgCl_2_, 1% Triton X-100 in PBS (pH 6.8) and then stained with Rhodamine conjugated isolectin B4 (dilution 1:100) overnight at 4 °C (Vector Laboratories Inc, Burlingame, CA, USA). To determine the extent of the avascular and neovascularization areas at P12, P14, and P17 we used the SWIFT-neovascularization plugin from ImageJ software (NIH, Bethesda, MD, USA) [30].

### Analysis of retinal vasculature

Imaging of whole retinas was done with a LSM800 confocal laser-scanning microscope (Zeiss) and using the automated tiling and stitching module of the ZEN Blue software. The number of tip-cells and the number of filopodia per tip-cell were quantified manually in a blinded manner. Quantification of tip-cell orientation, oriented between 60° and 120° relatively to the edge of the growing vascular front, was done using the angle tool of Zen Blue. Tip-cell morphology, normal or dystrophic, was quantified in a blinded manner. Dystrophic tip-cells are characterized by their bunted and rounded morphology.

### Single-cell RNA sequencing

Retinas were digested as in [31], and single-cell suspensions were prepared from P14 OIR eNOS^+/+^ (C57BL/6J) and eNOS^−/−^ mouse retinas (*n* = 3 replicates per genotype, with 3–5 retina pooled per replicate). The final EC suspension was obtained by positive enrichment using beads coated with CD31 antibodies and magnetic columns. For single-cell RNA-seq (scRNA-seq) of endothelial-enriched retinal cell suspension, droplet generation and cDNA libraries were performed as described in the Drop-seq procedure (http://mccarrolllab.org/dropseq/), and sequencing was carried out on an Illumina NextSeq 500 at an estimated read depth/cell similar to that used by Macosko et al. [31] (i.e., 50,000 reads/cell). Unique molecular identifier (UMI) counts associated with aligned reads (kb-python, GRCm38 reference genome) from the scRNA-seq replicates of eNOS^+/+^ and eNOS^−/−^ retina were merged into one single digital gene expression (DGE) matrix and processed using the “Seurat” package (spatial reconstruction of single-cell gene expression data [32]). Cells expressing fewer than 100 genes and more than 10% of mitochondrial genes were filtered out. Single-cell transcriptomes were normalized by dividing by the total number of UMIs per cell and then multiplying by 10,000. All calculations and data were then performed in log space [i.e., ln(transcripts per 10,000 + 1)].

After integration of the different biological replicates using the Seurat anchor integration algorithm [33], the 20 most significant components were used as input for dimensionality reduction and clustering. To identify putative cell types from the Uniform Manifold Approximation and Projection (UMAP) dimensional reductions, a graph-based clustering approach using *K*-nearest neighbor graph and the Louvain algorithm was used to define clusters and average gene expression was computed for each of the identified cluster based on Euclidean distances. Marker genes that were significantly enriched for each cluster were then identified, allowing cluster annotation to specific cell types. A similar computational approach was performed on non-integrated RNA data to sub-cluster the population of ECs, allowing us to define three cell subtypes based on marker genes. After removing the contaminant cell cluster (i.e., red blood cells and retinal pigmented epithelium), a total of 3031 cells were obtained from eNOS^+/+^ retina (including 2044 ECs) and 5913 cells from eNOS^−/−^ retina (including 3334 ECs). Transcriptomic differences between eNOS^+/+^ and eNOS^−/−^ EC types were statistically compared using a negative binomial model and analyzed using visualization tools including Dot Plot and heatmap plot.

For pathway analysis, normalized single-cell gene expression profiles from each distinct cell type identified by scRNA-seq were further analyzed using GSVA [34]. In contrast, direct pathways comparison based on transcriptomic differences between eNOS^+/+^ and eNOS^−/−^ ECs were analyzed using GSEA [35]. Fastq and raw count matrix are deposited on GEO DataSets (accession number GSE174400).

### Statistical analysis

Data are represented as the means ± SEM. Two-tailed independent Student’s *t*-tests were used when comparing two groups. Comparisons between multiple groups were made using two-way ANOVAs followed by post hoc Bonferroni’s multiple comparisons test among groups using GraphPad Prism 5 and R software. *p*-Value < 0.05 was considered statistically significant.

## Results

### eNOS knockdown prevents VEGF-induced migration and sprouting but promotes endothelial cell polarization

**Fig. 1:**
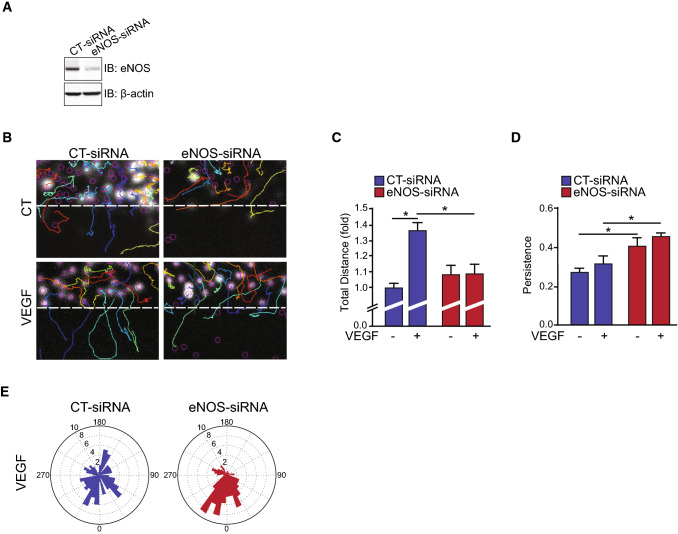
eNOS influences endothelial cell migration. Bovine arterial endothelial cells (BAECs) were transfected with control siRNA (CT-siRNA) or eNOS-siRNA to knockdown the expression of eNOS. **A** Depletion of eNOS was monitored by immunoblot (IB). β-actin was used as a loading control. **B** Confluent CT-siRNA or eNOS-siRNA transfected BAEC monolayers were scratched and tracked for 6 h in the presence or absence of 40 ng/mL VEGF. Tracks of nuclei are represented. **C**, **D** Quantifications of the total cell migration and persistence of displacements. Persistence is calculated by the ratio of the net (distance between the start and the final position) and the total displacement for each track. **E** Rose plot diagrams showing cell migration paths of CT-siRNA and eNOS-siRNA transfected BAECs. Results are displayed as mean values ± SEM. The graphs are representative of more than three independent experiments each yielding similar results. At least 60 cells per experiment were quantified. *p < 0.05

To investigate the role of eNOS expression on endothelial function, siRNA against eNOS (eNOS-siRNA) were used to knockdown the expression in bovine aortic endothelial cells (BAECs) prior to measurements of migration and polarization. eNOS-siRNA transfection markedly reduced eNOS protein expression compared to control-transfected (CT-siRNA) (Fig. 1A). Time-lapse in vitro wound healing assays were performed on these cells to examine the involvement of eNOS in the migration of BAECs (Fig. 1B). As expected, VEGF stimulation promoted the migration of ECs in CT-siRNA transfected cells (Fig. 1B, C). This migratory effect was completely blocked in cells with reduced eNOS expression. Persistency of the migratory movement, a measure of directionality, was not increased following VEGF stimulation in control ECs, indicating that VEGF is mostly inducing a non-directional type of migration (Fig. 1D). Interestingly, we observed that the residual migration of eNOS-depleted BAECs in the absence and presence of VEGF was more directional than the migration of CT-siRNA transfected ECs (Fig. 1D). This pattern of directional migration is reflected in rose plot diagrams showing that the cell migration paths of eNOS-siRNA transfected BAECs were largely perpendicular to the orientation of the scratch compared to control cells (Fig. 1E). These results indicate that eNOS plays a role in the polarization of ECs migration, which could regulate sprouting angiogenesis.

### eNOS is a regulator of polarity in endothelial cells

**Fig. 2:**
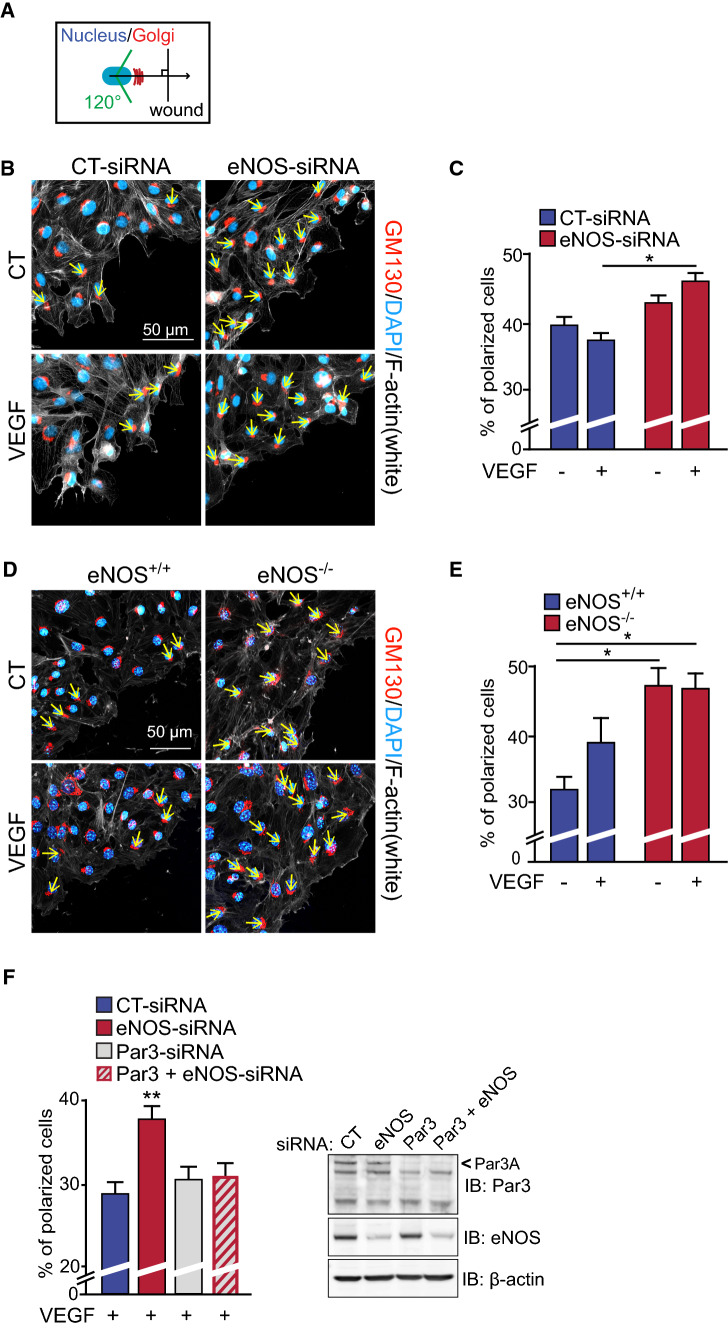
eNOS expression influences the polarization state of endothelial cells. **A** Schematic representation of Golgi apparatus orientation measurements during a wound healing migration assay of confluent cell monolayers. Cells were subsequently fixed and stained for GM130 (Golgi) (red) and the nucleus (blue). Positions of the nucleus (blue) and the Golgi (red) are represented as a function of the position of the wound. The number of orientated cells was quantified in the first 2–3 layers and reported relative to the total number of cells in those layers. **B** Confluent CT-siRNA and eNOS-siRNA transfected BAECs were scratched and treated with VEGF as indicated for 30 min. The leading edge of cells at the migration front was made visible by F-actin staining (phalloidin, white). Representative images are shown. **C** Quantification of polarized transfected BAECs. **D** Mouse lung endothelial cells (MLECs) derived from eNOS^−/−^ mice and controls eNOS^+/+^ were scratched and treated with VEGF for 30 min where indicated. **E** Quantification of polarized MLECs is shown. Results are displayed as mean values ± SEM. *p < 0.05. The graphs are representative of three independent experiments yielding similar results. **F** Quantification of polarized VEGF-stimulated BAECs that were transfected with CT-siRNA, eNOS-siRNA, Par3-siRNA or both Par3- and eNOS-siRNA. Depletion of eNOS and Par3 were monitored by immunoblot (IB). β-actin was used as a loading control. At least 50 cells and 25 cells per condition were quantified for BAECs and MLECs, respectively. *p < 0.05

To further investigate the role of eNOS in EC polarization, we examined the orientation of the Golgi apparatus, a marker of EC polarity, in BAECs transfected with CT- and eNOS-siRNA during wound healing assays of confluent cell monolayers. Golgi orientation, as a function of its position relative to the nucleus and perpendicular to the wound border (Fig. 2A), was quantified on cells positioned directly behind the scratch following 30 min of VEGF stimulation (Fig. 2B, C). Similar to the directionality of cell migration, VEGF stimulation of CT-siRNA-transfected ECs did not induce a significant orientation of the Golgi towards the scratch edge providing further evidence for induction of non-orientated migration of ECs following VEGF stimulation (Fig. 2B, C). However, eNOS-depleted ECs displayed greater orientation of the Golgi towards the leading edge in response to VEGF, compared to VEGF-stimulated CT-siRNA transfected BAECs (Fig. 2C).

Golgi orientation during VEGF-stimulated EC migration was also examined in mouse lung ECs (MLECs) isolated from eNOS knockout (eNOS^−/−^) and age-matched wildtype C57Bl/6J mice (eNOS^+/+^) (Fig. 2D, E). Interestingly, unstimulated eNOS^−/−^ MLECs had increased basal levels of Golgi orientation toward the leading edge compared to MLECs from eNOS^+/+^ mice. VEGF stimulation of eNOS^−/−^ and of eNOS^+/+^ ECs did not affect further Golgi polarization during cell migration (Fig. 2E). Moreover, comparable results were obtained in BAECs where eNOS activity was inhibited using the NOS inhibitor L-NMMA (Supplemental Fig. S1). These results uncover a role for eNOS in cell polarization of ECs.

These results prompted us to examine the involvement of PAR polarity proteins in the polarization of the Golgi induced by the downregulation of eNOS in ECs. We examined the effects of siRNA-mediated downregulation of Par3 on Golgi polarization in eNOS-downregulated ECs. As above, downregulation of eNOS resulted in increased orientation of the Golgi towards the edge of the wound in VEGF-stimulated BAECs (Fig. 2F). Downregulation of Par3 alone (Par3-siRNA) did not affect Golgi orientation compared to control BAECs. However, co-transfection of cells with Par3-siRNA and eNOS-siRNA prevented the effects of eNOS depletion on Golgi orientation. This suggests that the polarity protein Par3 is necessary for the effects of eNOS downregulation on polarization of ECs.

### eNOS is important for endothelial cell sprouting

**Fig. 3:**
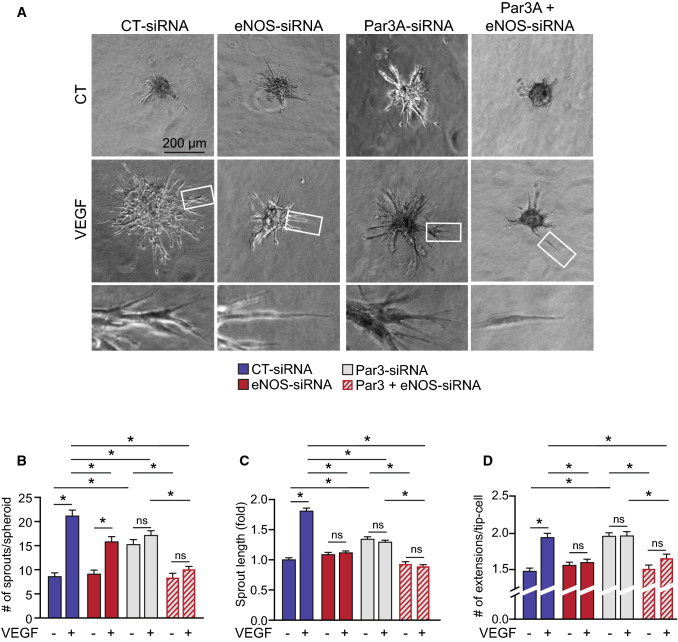
eNOS is important for VEGF-induced endothelial cell sprouting. **A** Representative images from spheroid-based angiogenesis assays generated from BAECs transfected with control siRNA (CT-siRNA), eNOS-siRNA, Par3-siRNA or both (Par3 + eNOS-siRNA) and exposed to VEGF (40 ng/mL) or left untreated. Pictures were taken 24 h after embedding in a collagen matrix. **B** Quantification of the number of capillary-like sprouts from spheroids was measured following VEGF stimulation. **C** The length of sprouts was measured and expressed relatively to spheroids that were not stimulated with VEGF. **D** The number of extensions per tip-cell was quantified. Data are represented as mean ± SEM of three experiments with at least 8 spheroids per condition per experiment. *p < 0.05

To validate the functional implications of eNOS in angiogenic sprouting, we used a 3-dimensional spheroidal system of EC sprouting. EC spheroids were set in collagen gel and the outgrowth of capillary-like structures was analyzed following VEGF treatment of BAECs transfected with CT-siRNA or eNOS-siRNA (Fig. 3A). VEGF stimulation of control-transfected cells resulted in an increase in the number of sprouts per spheroid as well as the length of those sprouts (Fig. 3B, C). These effects of VEGF were abolished in eNOS-depleted ECs. We also found that tip-cells at the extremity of sprouts in eNOS-depleted spheroids had fewer cell extensions, reminiscent of filopodia, in response to VEGF compared to CT-siRNA spheroids (Fig. 3D). These results further support the importance of eNOS in the induction of angiogenic sprouting by VEGF.

We also examined the contribution of Par3 to eNOS-regulated EC sprouting. Interestingly, downregulation of Par3 in ECs induced their spontaneous sprouting from unstimulated spheroids (Fig. 3A). The number and length of sprouts as well as the number of extensions per tip-cell were basally increased in Par3-siRNA transfected ECs compared to CT-siRNA (Fig. 3B–D). Sprouting and extensions per tip-cell from Par3-downregulated spheroids were not enhanced further by VEGF treatment. Dual transfections of ECs with Par3-siRNA and eNOS-siRNA significantly reduced VEGF-stimulated sprouting from spheroids and formation of extensions from tip-cells (Fig. 3A–D). Additionally, the unstimulated basal sprouting seen in Par3-depleted spheroids was abolished in spheroids co-transfected with Par3- and eNOS-siRNA. Overall, this series of experiments show that inhibition of eNOS blocks VEGF-stimulated EC migration and sprouting but enhances cell polarization. The polarization of eNOS-inhibited ECs is dependent on the expression of Par3.

### eNOS affects the expression of key polarity genes in endothelial cells

To understand how eNOS expression could regulate EC polarization, orientated migration and angiogenic sprouting, we compared the transcriptomes of control and eNOS-depleted BAECs. RNA-seq was carried out on BAECs transfected with either CT-siRNA or eNOS-siRNA. eNOS levels were knocked down by more than 80% in eNOS-siRNA transfected cells (Fig. 1A). The sequencing yielded more than 40 million reads from each sample and ~ 14,000 genes were mapped from these reads. The number of reads assigned for each gene was used to calculate the gene differential expression. Thresholds of fold change > 1 log2 fold and < -1 log2 fold were set to determine the regulated genes in each analysis (Supplemental Fig. S2). First, the RNA-seq analysis confirmed that the mRNA levels of bovine *NOS3*, the gene that encodes eNOS, were reduced by more than 85% in eNOS knockdown cells compared to the control BAECs. Overall, a comparison of control siRNA and eNOS-siRNA transfected BAECs revealed that 1542 genes were differentially regulated, including 808 upregulated and 734 downregulated mRNAs in eNOS knockdown BAECs.

**Fig. 4:**
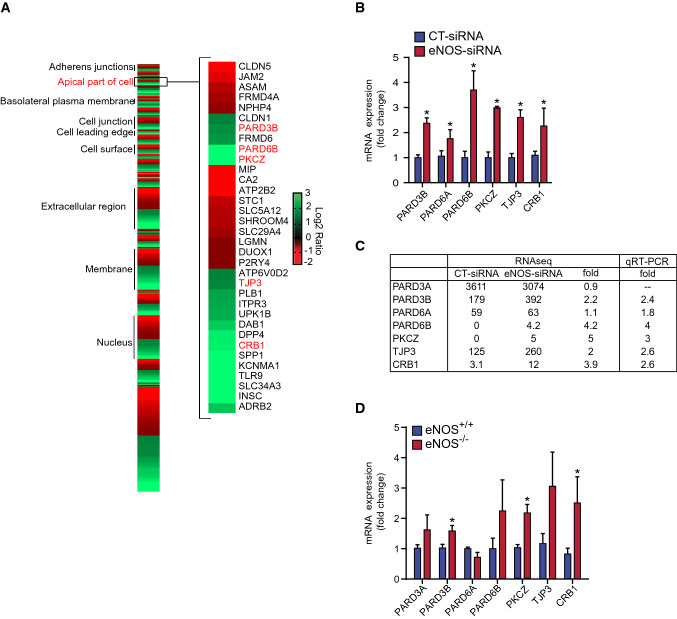
eNOS down-regulation increases the expression of several polarity genes. **A** Heat map showing the cellular component of eNOS regulated gene products obtained by RNA-seq analysis. BAECs were transfected with CT-siRNA or eNOS-siRNA. RNA was then extracted and submitted for RNA-Sequencing. The reads were aligned to the UMD3.1 genome with TopHat, using the Ensembl annotation. A threshold of fold change > 2 or < 0.5 was used to define the regulated genes. These genes were submitted to the DAVID database and categorized using gene ontology cellular component annotation. Genes composed of the “apical part of cell” (GO: 0045177) are shown. **B** qRT-PCR validation of genes of interest identified from the RNA-seq analysis of siRNA transfected BAECs. Gene expression analysis was performed using the comparative cycle threshold method, normalized using reference gene GAPDH expression and presented as the mean fold change (± SEM) compared with control. Results are averages of three independent experiments, each performed in quadruplicates. **C** Summary table of RNA-seq and qPCR results. **D** Mouse lung endothelial cells (MLECs) from wildtype (eNOS^+/+^) and eNOS^−/−^ mice were cultured, and expression of polarity genes were measured by qRT-PCR, normalized to GAPDH or β-actin housekeeping genes, and expressed as fold change of eNOS^+/+^ cells. Results are averages of four independent experiments, each performed in quadruplicates. Results are displayed as mean values ± SEM. *p < 0.05

To characterize the eNOS regulated transcriptomes in BAECs, genes from affected mRNAs were grouped into their respective GO annotations, including biological process, cellular component and molecular function (Supplemental Fig. S3). Interestingly, the biological processes annotations locomotion (GO: 0040011), cell motility (GO: 0048870) and cell adhesion (GO: 0007155) as well as the cellular component annotations apical junction complex (GO: 0043296), extracellular region part (GO: 0044421) and cell junction (GO: 0005911) were significantly enriched. Furthermore, eNOS-regulated genes were found to be enriched within the GO term Apical Part of Cell (GO: 0045177) (Fig. 4A), which is related to cellular polarization. These encompass several genes involved in cell polarity, including the crumbs (CRB) family of EGF-like protein 1 (CRB1), TJP3, PKCZ, and Partitioning Defective (PARD) family proteins. The regulation of a selection of these genes was confirmed by qRT-PCR (Fig. 4B). PARD3B, PARD6A, PARD6B, PKCΖ, TJP3, and CRB1 were all significantly upregulated in eNOS-depleted BAECs. A comparison table of results obtained from RNA-seq and qPCR in BAECs transfected with CT- and eNOS-siRNA is shown in Fig. 4C. Moreover, qPCR of MLECs isolated from eNOS^−/−^ and wildtype (eNOS^+/+^) showed that, as in the BAEC model, eNOS^−/−^ cells expressed significantly higher levels of PARD3B, PKCZ, and CRB1 than eNOS^+/+^ (Fig. 4D). eNOS depletion appears to affect EC polarization by upregulating the expression of polarity genes of ECs.

### eNOS is an essential mediator of retinal angiogenesis

**Fig. 5:**
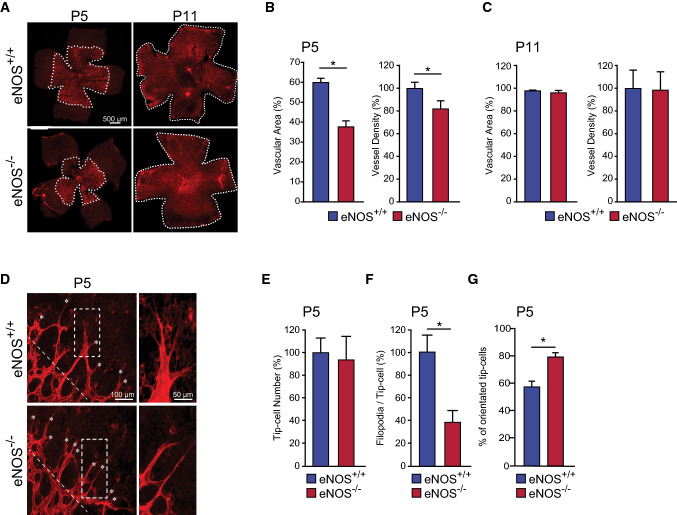
eNOS is an essential mediator of retinal angiogenesis. **A** High-resolution imaging of Isolectin B4–stained mouse retina at postnatal day 5 (P5) and P11 from eNOS^+/+^ and eNOS^−/−^ mice. The percentage of the vascular area and vessel density was measured in **B** P5 (*n* = 7 per group) and **C** P11 (*n* = 9 per group) retinas from eNOS^+/+^ and eNOS^−/−^ mice. **D** Representative images from P5 retinas showing tip-cells and filopodia. The numbers of **E** tip-cells, **F** filopodia per tip-cell, and **G** the percentage of oriented cells (cells pointing in between 60° and 120° perpendicular to the vascular front) were determined in retinas from eNOS^+/+^ and eNOS^−/−^ mice (*n* = 10 retinas per group). White dashed lines in **D** indicates the orientation of the vascular front. Results are displayed as mean values ± SEM. *p < 0.05

To further explore the role of eNOS in sprouting angiogenesis during vascular development, we investigated the role of eNOS during the development of retinal vascularization in eNOS^−/−^ and eNOS^+/+^ mice. Retinas from eNOS^−/−^ mice at post-partum day 5 (P5) displayed a decrease in the vascularized area and vessel density compared to retinas from eNOS^+/+^ mice (Fig. 5A, B). This indicates a role of eNOS as a modulator of angiogenic sprouting during vascular development in vivo. Further, we confirm previous reports showing that at maturity (P11) the vascular area and density of retinas from eNOS^−/−^ mice do not differ from eNOS^+/+^ mice (Fig. 5C) [36]. These results indicate that eNOS plays a role in the growth of developing retinal blood vessels in the mouse retina.

To explain the delay in retinal vascular development in eNOS deficient mice, we examined the morphology of the highly motile tip-cells located at the front of the developing retinal microvasculature (Fig. 5D). While the number of tip-cells located at the extremities of the vascular front was similar in eNOS^−/−^ and wildtype (Fig. 5E), tip-cells in eNOS^−/−^ retinas at P5 had a marked reduction in the number of filopodia per cell (Fig. 5F). Furthermore, the percentage of tip-cells that are oriented perpendicular (between 60° and 120°) relative to the edge of the growing vascular front was significantly higher in eNOS^−/−^ mice (Fig. 5G). These results corroborate our in vitro 3-dimensional angiogenesis assays (Fig. 3) suggesting that polarization, and consequently orientated migration of tip-cells are increased during angiogenic sprouting in eNOS^−/−^ retinas.

### eNOS modulates pathological retinal neovascularization

**Fig. 6:**
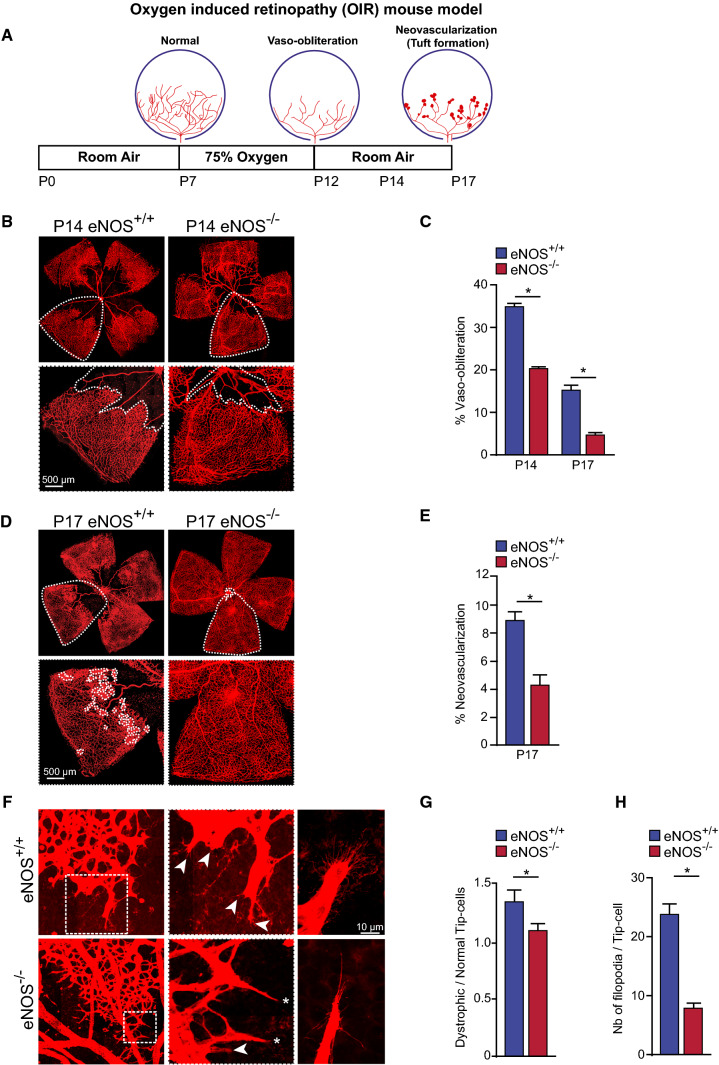
eNOS is a modulator of pathological neovascularization in oxygen-induced retinopathy. **A** Schematic representation of oxygen-induced retinopathy (OIR) mouse model. From postnatal day 7 (P7) to P12, mice are exposed to hyperoxic conditions (75% O_2_), leading to vaso-obliteration. Mice are then returned to room air resulting in hypoxia-driven revascularization and pathologic neovascularization, and tuft formation. **B** Representative high-resolution imaging of Isolectin B4–stained mouse retina at P14. **C** Quantification of vaso-obliteration (VO) is expressed as the central avascular areas divided by the total area of the retina for each strain. **D** Representative high-resolution imaging of Isolectin B4–stained mouse retina at P17. Areas of neovascularization are outlined in lower panels. **E** Quantification of neovascularization area in mouse strains. **F** Representative images of normal (asterisks) and dystrophic cells (arrowheads) at the vascular front of the retina in eNOS^+/+^ and eNOS^−/−^ mice. **G** Quantification of the ratio of dystrophic tip-cells to normal tip-cells. **H** Measurement of the number of filopodia per tip-cell. Results are displayed as mean values ± SEM. *p < 0.05 (*n* = 6 for P14 and *n* = 10 for P17 eNOS^+/+^ mice; *n* = 22 for P14 and *n* = 34 for P17 eNOS^−/−^ mice)

To investigate the role of eNOS in pathological retinal angiogenesis, we took advantage of the widely used mouse model of Oxygen Induced Retinopathy (OIR). Classically, OIR progresses in two phases: an initial vaso-obliterative phase of immature vessels followed by pathological neovascularization during the subsequent vascular repair phase [37]. Since eNOS^−/−^ mice display reduced speed of vascular sprouting during retinal vascular development, we hypothesized that deletion of eNOS would result in reduced pathological neovascularization. eNOS^+/+^ and eNOS^−/−^ mouse litters were placed in 75% oxygen for 5 days starting at P7 then were returned to room air until P12. The retinas were collected at P14, and P17 (Fig. 6A).

Vaso-obliterated (VO) areas of the retina were assessed by measuring the area devoid of vasculature over the total flat-mounted retinal area; blood vessels were stained with isolectin-B4 [30, 38]. At P14 and P17, eNOS deficient mice had markedly reduced vaso-obliteration compared to eNOS^+/+^ mice (Fig. 6B, C). Remarkably, at P17, the VO areas were almost completely revascularized in eNOS^−/−^ mice (Fig. 6D, E). Preretinal tufts or pathologic neovascularization at P17 that protrude into the vitreous were analyzed and quantified using the SWIFT-NV computer-aided quantification method [30]. eNOS^−/−^ retinas experience a significant regression of the pathological neovascularization compared to eNOS^+/+^ controls (Fig. 6D, inset).

Angiogenic sprouting is promoted by active filopodial protrusions and tip-cell migration [39]. To further characterize the pathological neovascularization of these mouse strains we examined the morphologic shape of tip-cells at P14. The ratio of dystrophic to normal tip-cells at the vascular front was lower in eNOS^−/−^ mice compared to controls at P14 (Fig. 6F, G), suggesting that deletion of eNOS reduces the pathological tip-cell morphology in the retina. Further, tip-cells in eNOS^−/−^ mice expressed fewer filopodia per tip-cell than eNOS^+/+^ controls (Fig. 6F, H), indicating a higher level of polarization. Collectively, these results suggest that the efficient revascularization of eNOS^−/−^ retinas could be due to the increased polarization of ECs, which protects from hyperoxia-induced VO by promoting remodelling of the retinal vasculature at a faster rate.

### eNOS deficiency activates cell polarity pathways in pathological retinal neovascularization

**Fig. 7:**
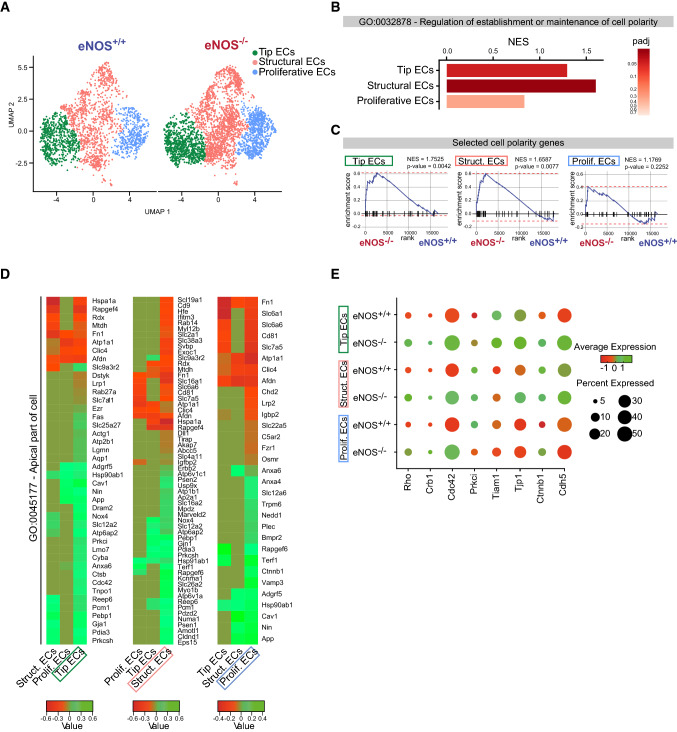
Single-cell RNA-sequencing analysis of ECs of retinas from eNOS^−/−^ mice compared to WT at P14 of OIR. **A** Uniform Manifold Approximation and Projection (UMAP) analysis and representation of single-cell gene expression of P14 OIR retinas from eNOS^+/+^ and eNOS^−/−^ (*n* = 4 per group) shows three identified EC subpopulations (tip, structural and proliferative). **B** Bar graph of the Normalized Enrichment Score (NES) of the ¨GO: 0032878 Regulation of establishment or maintenance of cell polarity¨ in tip, structural and proliferative ECs. **C** Gene set enrichment analysis (GSEA) of curated cell polarity gene signature in P14 OIR eNOS^-/-^ and eNOS^+/+^ retina in tip, structural and proliferative ECs (*p*-value and NES of each GSEA are indicated). **D** Heatmaps of the ¨GO: 0045177 Apical Part of Cell¨ showing enrichment in deficient eNOS P14 OIR retinas in the three sub-populations of ECs. **E** Dot plot demonstrates enrichment of polarity genes expression in the three EC subpopulations eNOS^−/−^ compared to eNOS^+/+^. Dot size corresponds to proportion of cells within the group expressing each transcript, and dot color corresponds to expression level (green high and red low expression)

To understand how eNOS expression affects the retinal vasculature during hyperoxia-induced retinopathy, we used single-cell mRNA transcript analysis (scRNA-seq) by Drop-Seq [31] to determine the differential expression of genes in EC subtypes of retinas from eNOS^−/−^ mice compared to WT at P14 of OIR. ECs from dissociated retinas of eNOS^−/−^ and eNOS^+/+^ mice were enriched by CD31 purification and the scRNA-seq data were analyzed to classify individual cells into cell subpopulations according to similarities in their transcriptome profiles. Overall, the cells were classified into ten transcriptionally distinct clusters (Supplemental Fig. S4A). We focused mainly on the EC cluster (Pecam1/CD31 and Cldn5 enriched cells) (Supplemental Fig. S4B). Unsupervised analysis identified three sub-clusters in the EC population, namely Tip ECs, Structural ECs and Proliferative ECs (Fig. 7A).

The Tip ECs cluster displayed a vascular endothelial signature with increased expression of specific tip-cell genes including already known Angiopoietin 2 (Angpt2), adrenomedullin (Adm) and Apelin (Apln) [40, 41] and recently identified tip-cell markers Trp53i11 and Carbohydrate sulfotransferase 1 (Chst1) (Supplemental Fig. S4C) [42, 43]. Proliferative ECs displayed overexpression of cell cycle genes such as Top2a and Mki67 and structural cluster did not express tip or proliferation cell markers (Supplemental Fig. S4C). The relative proportion of EC subtypes quantified by scRNA-seq was not significantly different between eNOS^+/+^ and eNOS^−/−^ in P14 ischemic retinas (Supplemental Fig. S4D). Table S1A shows the top differentially expressed genes (DEGs) per cluster between eNOS^−/−^ and eNOS^+/+^ sorted by mean expression and log2 fold change. The complete dataset is presented in Table S1B.

We set out to examine comprehensively the gene expression signatures related to cell polarity in the three sub-populations of eNOS deficient retinal ECs compared to WT. Hence, differential expression analysis within each EC subpopulation was performed by comparing the level of expression of polarity genes in EC subtypes originating from eNOS ^−/−^ and eNOS^+/+^. Gene set enrichment analysis (GSEA) of the GO term “Regulation of establishment or maintenance of cell polarity” (GO: 0032878) was markedly enriched in Structural ECs (Normalized Enrichment Score (NES) = 1.5997; *p*-value = 0,024) followed by the Tip ECs subpopulation (NES = 1.3570; *p*-value = 0,041) and was not significantly enriched in Proliferative ECs (NES = 0.8340; *p*-value = 0.7175) (Fig. 7B). GSEA using curated polarity genes from different polarity complexes pathways (listed in Table S2) between eNOS ^−/−^ and eNOS^+/+^ showed a significant enrichment of these polarity genes in Tip ECs (NES = 1.7525; *p*-value = 0.0042) and to a lesser extent in Structural ECs (NES = 1.6587; *p*-value = 0.0072) and non-significant enrichment was found in proliferative ECs (NES = 1.1769, *p*-value = 0.2252) (Fig. 7C). Moreover, significant DEGs between eNOS^−/−^ and eNOS^+/+^ that are part of the GO term “Apical part of the cell” (GO: 0045177) are shown in heatmaps for each EC subtype (Fig. 7D). These analyses revealed an increased expression of known polarity genes in Tip ECs, namely Cdc42, Prkci (Protein Kinase C Iota) and Cav1 (caveolin 1) and also the newly demonstrated polarity gene Gja1, also known as connexin 43 (Cx43) [44–46].

Finally, specific analysis for the polarity genes Rho, Crb1, Prkci, Cdc42 and Tiam1 as well as the tight junction protein ZO-1 (Tjp1), and the adherens junction proteins β-catenin (Ctnnb1) and cadherin 5/VE-cadherin (Cdh5) showed higher mRNA expression levels in eNOS deficient OIR ECs compared to WT. This difference was particularly obvious in Tip and Structural ECs of ischemic retinas (Fig. 7E). Overall, these results demonstrate that eNOS regulates in retinal ECs genes associated with cell polarization in a murine model of proliferative retinopathy.

## Discussion

During the progression of OIR, uncontrolled angiogenesis leading to pathological neovascularization, can often cause irreparable loss of visual acuity. Therapeutic strategies to control these poorly understood angiogenic processes in this setting would prove extremely useful in combating ischemic retinopathies. This study further elucidated the role of eNOS in critical angiogenic processes including cell polarity, migration, and transcriptional regulation of angiogenesis-relevant proteins. Additionally, the effect of eNOS on the vaso-obliteration and neovascularization phases of ischemic retinopathy was examined.

While it was previously known that eNOS is involved in retinal angiogenesis how eNOS regulates this process remained unclear. Herein, we show that eNOS controls endothelial cell polarization and affects the expression of endothelial cell polarity genes. We further show that eNOS knockdown prevents VEGF-induced migration and sprouting revealing the role of eNOS in endothelial cells during sprouting angiogenesis. In the context of pathological neovascularization, we showed that eNOS deficiency induces cell polarity pathways, which may decrease the propensity of migratory tip-cells to be misguided by excessive VEGF present in the ischemic retina. In addition to VEGF, other endothelial growth factors, such as angiopoietins, are involved in the regulation of normal and pathological retinal angiogenesis [47, 48]. Angiopoietin-1 is well-known to induce vessel stabilization, directed EC migration and to inhibit VEGF-induced vascular permeability and eNOS activation [28, 47, 49, 50]. Interestingly, intravitreal administration of angiopoietin-1 was shown to prevent vascular degeneration in mouse models of retinopathies [47, 51].

The role of eNOS on retinal blood vessel development has previously been investigated using animal models with modified eNOS expression. In eNOS^−/−^ mice, vascular density, patterning and individual vessel length were normal at day P30 [36]. Surprisingly, NO levels in the eNOS^−/−^ retinas did not differ from eNOS^+/+^ mice, suggesting that neuronal NOS (nNOS) produced in other retinal layers could compensate for the loss of eNOS, preserving normal vessel density once the development of the retinal vasculature is completed [36]. Here, we confirm that at maturity (P11) and under normoxic conditions the vascular area and density of retinas from eNOS^−/−^ mice are not different from WT mice (Fig. 5C). However, early vascular development of the retina is delayed at P5, and blood vessel density is decreased in eNOS^−/−^ mice (Fig. 5B).

We have also confirmed previous reports that eNOS^−/−^ mice are protected from vaso-obliteration in response to hyperoxia (P14 OIR), suggesting enhanced physiological revascularization of the retina [20]. Recent studies also demonstrate that inhibition of eNOS activity and expression in mice reduces vascular leakage by stabilizing endothelial adherens junctions of ECs in the vascular tufts contributing to the reduced pathological neovascularisation [25]. Noteworthy, the authors also showed that acute eNOS inhibition decreased vascular leakage without affecting overall blood perfusion of the retina. This study confirmed the direct actions of eNOS-derived NO on endothelial adherens junctions, which contributes to retinal vascular diseases [23, 24]. Our results add a new layer of complexity to the pleiotropic actions of eNOS in pathological angiogenesis by demonstrating that by directly affecting the polarity program of ECs, eNOS deletion leads to less misguided pathological neovascularization or tufts invading the vitreous body. In support of our observations, it has been shown that transgenic overexpression of eNOS resulted in increased severity of vaso-obliteration (a decrease in vascular closure during the obliterative phase) and exacerbated the formation of pathological neovascular tufts, which correlated with increased vascular branching [21]. Hence, increased tip-cell polarity in eNOS-depleted retinas might promote migratory persistence and the directional revascularization of the avascular retina while preventing misguided vessels from growing astray towards the vitreous body. Collectively, these results suggest further studies aimed at targeting eNOS as a strategy to avert retinal neovascularization are warranted.

Depending on the setting, NO exerts either an angiogenic [8, 52–54] or an antiangiogenic effect [55]. Further evidence indicating a context-dependent effect of NO comes from a study using targeted deletions of the three isoforms of NOS; it was shown that mice deficient in eNOS, but not iNOS or nNOS, experienced reduced OIR induced neovascularization [7]. However, in mice with laser-induced rupture of Bruch’s membrane, eNOS deficiency did not affect choroidal neovascularization, but lack of iNOS or nNOS caused a significant decrease in neovascularization [7]. Thus, the effect of NO is extremely context-dependent. In the context of OIR, to revascularize the developing retina following vaso-obliteration, NO is essential to quickly initiate angiogenesis but is critical to undergo this angiogenic process in a tightly controlled and 2-dimensional manner that does not infringe on the vitreous cavity. Our findings suggest that the absence of eNOS reduces the pathological neovascularization due to directed revascularization on the surface of the retina instead of the avascular vitreous. eNOS deficiency seems to lead to a polarized blood vessel growth on the superficial layer of the retina.

We conclude that eNOS plays an essential role as a regulator of EC polarity, regulating vascular regeneration and pathological neovascularization during retinopathy. To our knowledge, this is the first study to specifically examine eNOS regulation of polarity gene expression at the single endothelial cell level. We report a novel role for eNOS in retinal vascular endothelial cell polarity and vascular remodeling in the retina. Thus, our study may provide insight into a general mechanism in which eNOS may regulate EC polarity and directional cell migration needed for angiogenesis and vascular regeneration in other tissues and ischemic diseases.

## Supplementary Information

Below is the link to the electronic supplementary material.

Supplementary file1 (XLSX 102 KB)

Supplementary file2 (XLSX 10 KB)

Supplementary file3 (PDF 2048 KB)

## Data Availability

Fastq and raw count matrix of the scRNA-seq data are deposited on GEO DataSets (accession number GSE174400). All material and data are available upon request.
