# Supplementary material for: eNOS controls angiogenic sprouting and retinal neovascularization through the regulation of endothelial cell polarity
Source: Cell Mol Life Sci. 2021 Dec 31;79(1):37. doi: 10.1007/s00018-021-04042-y (PMC8739159; doi:10.1007/s00018-021-04042-y)

SUPPLEMENTARY FIGURE LEGENDS

Figure S1: Confluent BAEC pre-treated with L-NMMA (eNOS inhibitor) were scratched and stimulated with VEGF. Representative images shown. The line represents the leading edge. Percentage of polarized BAEC were quantified based on Golgi polarization (GM130 staining in red and DAPI in blue) in all conditions. Results are displayed as mean values ± SEM. * $p<0.05$, n = 10 per group)

Figure S2: Histogram showing the distribution pattern of the expression of all genes identified in the RNA-seq analyses comparing mRNA expression of CT-siRNA and eNOS-siRNA transfected BAECs. Dotted lines indicate the thresholds (>1 log2 fold and < -1 log2 fold) used to define the up- and downregulated genes.

Figure S3: GO enrichment analysis of genes regulated by eNOS knockdown and a heat map showing fold changes of selected genes. Fisher Exact p-value was used to define the significantly enriched GO terms. The GO terms with values –log10 (p-value) > 1.3 are considered significantly enriched (p-value < 0.05).

Figure S4: (A) Uniform Manifold Approximation and Projection (UMAP) analysis and representation of cell populations identified in p14 OIR retinas from eNOS$^{+/}$+ and eNOS (n = 4 per group). (B) UMAP presentation of specific expression of Endothelial cell marker (CD31 and Cldn5). (C) Dot plot demonstrates the top markers of each EC sub-type. Dot size corresponds to the proportion of cells within the group expressing each transcript, and dot color corresponds to expression level (red high and blue low expression). (D) Bar graphs show the number of ECs in each cluster of eNOS$^{-/-}$ deficient and eNOS$^{+/+}$ retinas.

Supplemental Table S1: Differentially expressed genes (DEGs) per cluster between $eNOS^{-/-}$ and $eNOS^{+/+}$.

Supplemental Table S2: List of selected polarity genes from different polarity complexes pathways used for Figure 7C.

Supplemental Figure S1

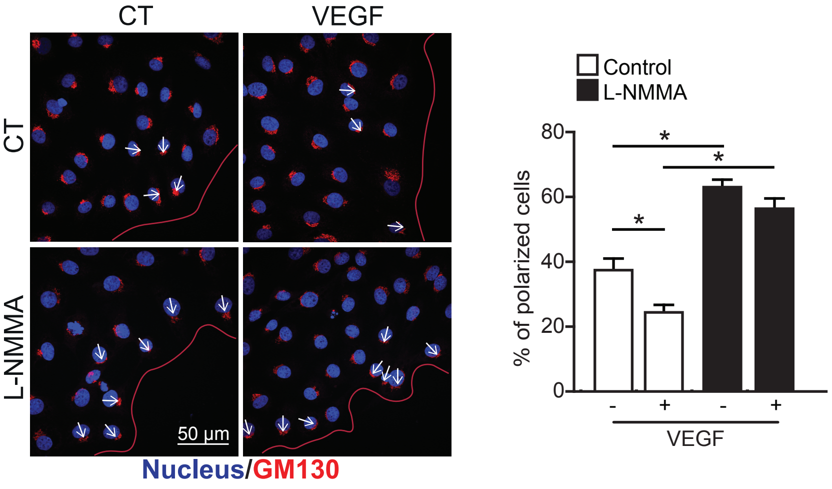

Supplemental Figure S2

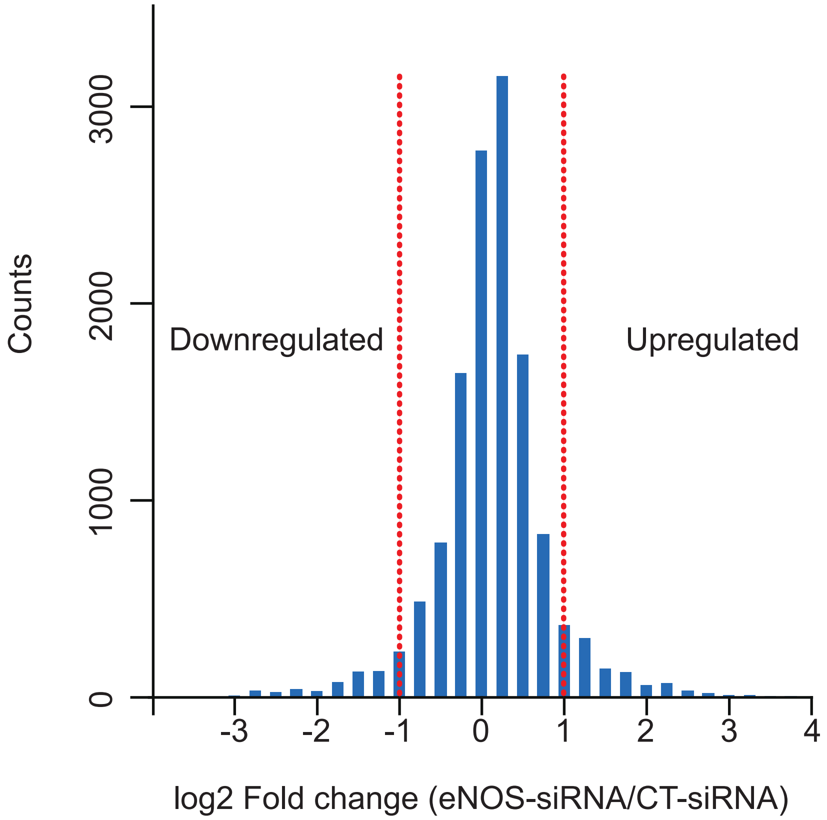

Supplemental Figure S3

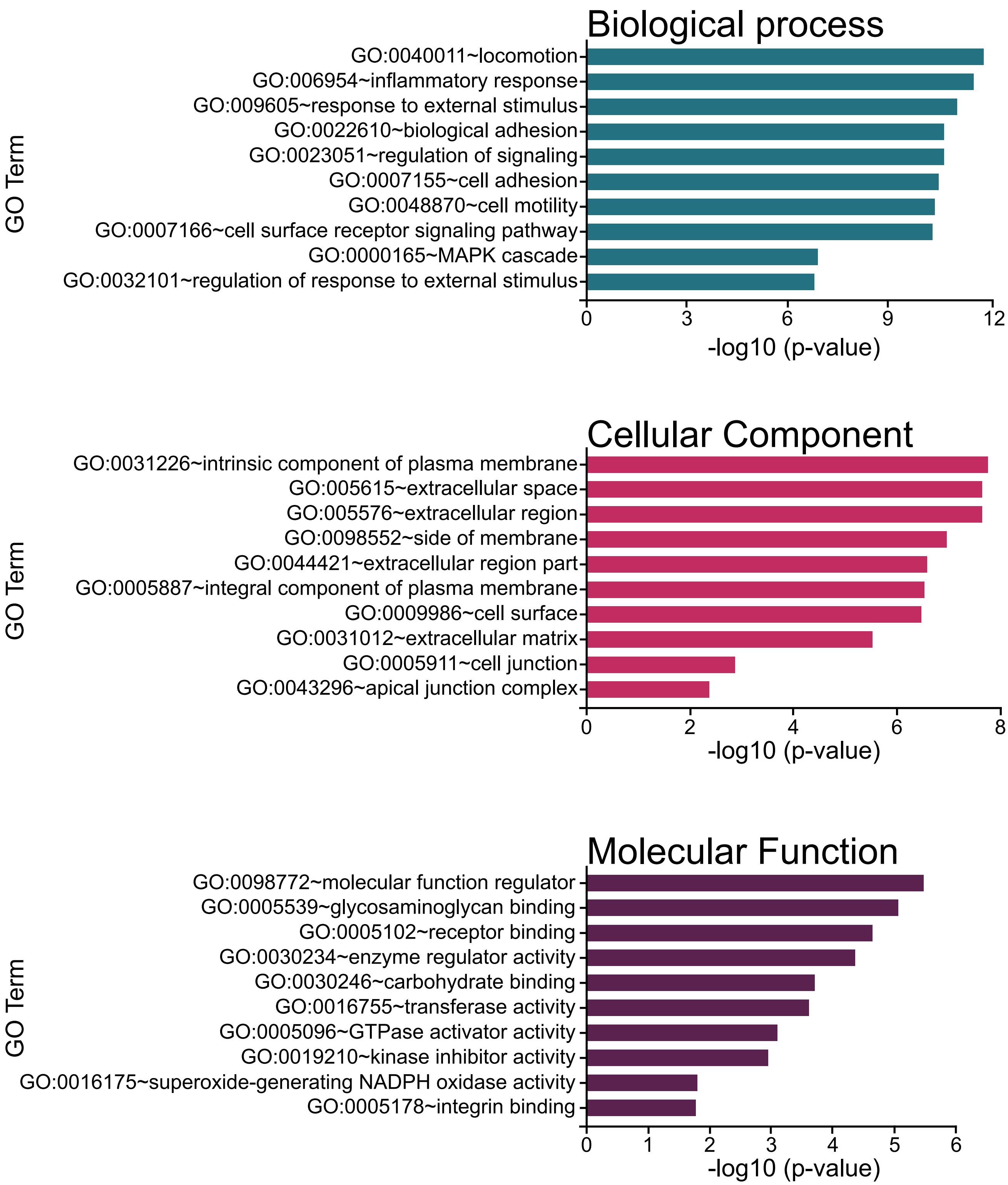

Supplemental Figure S4

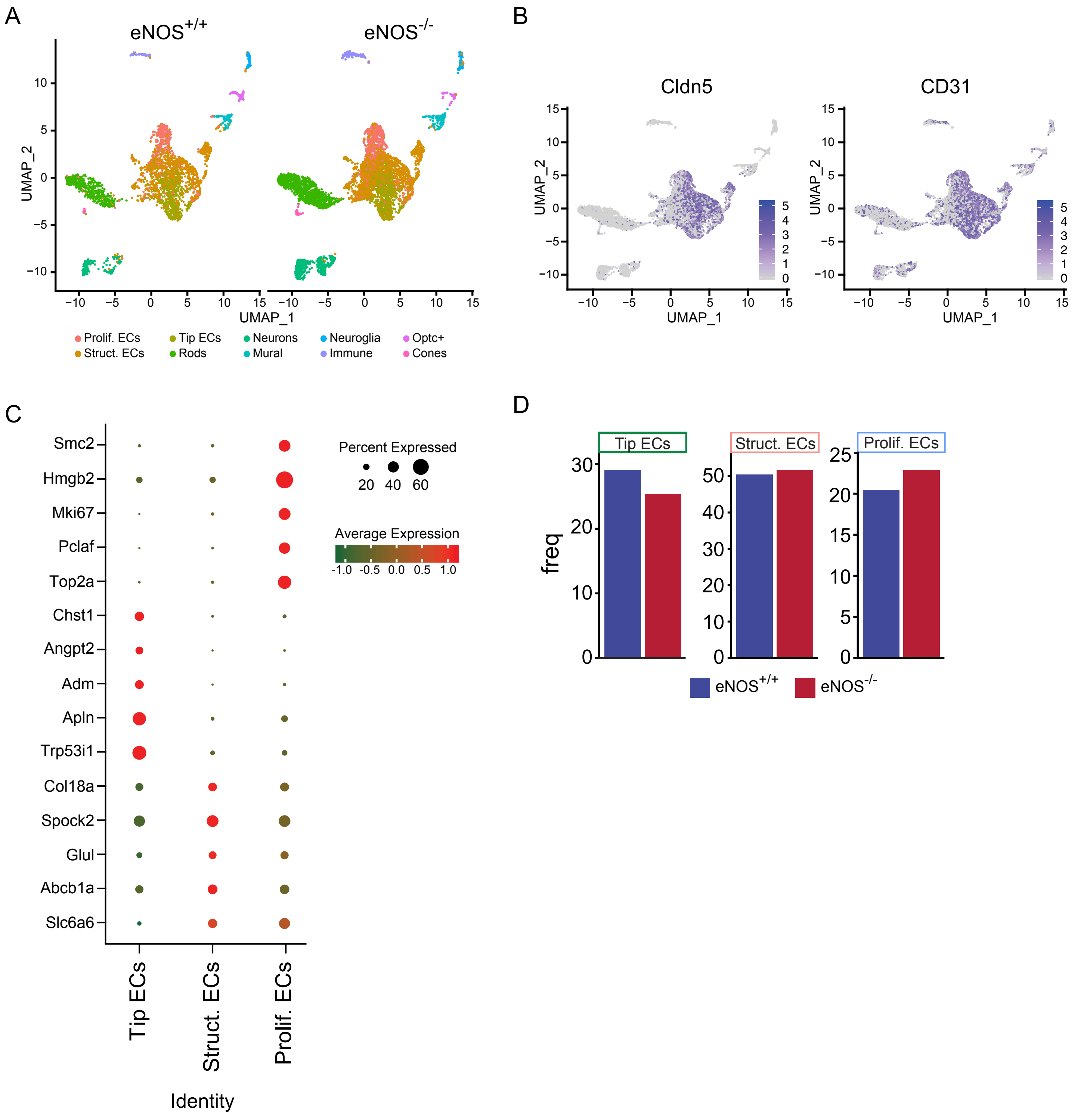

Supplement: Supplementary file 3 — Supplementary file3 (PDF 2048 KB) [file 18_2021_4042_MOESM3_ESM.pdf]
